# Efficacy and safety of endoscopic ultrasound-guided drainage of pancreatic pseudocysts using double-pigtail plastic stents: A single tertiary center experience

**DOI:** 10.6061/clinics/2021/e2701

**Published:** 2021-07-26

**Authors:** Giovana Biasia de Sousa, Rodrigo Strehl Machado, Frank Shigueo Nakao, Ermelindo Della Libera

**Affiliations:** Departamento de Endoscopia, Hospital Universitario, Universidade Federal de Sao Paulo (UNIFESP), Sao Paulo, SP, BR.

**Keywords:** Pancreatic Pseudocyst, Endoscopy, Drainage, Endosonography

## Abstract

**OBJECTIVES:**

Pancreatic pseudocysts (PPC) are fluid collections with a well-defined wall that persist for more than 4 weeks inside or around the pancreas as a result of pancreatic inflammation and/or a ductal lesion. PPC have been successfully treated with endoscopic ultrasound (EUS)-guided drainage using different stents. This study aimed to evaluate the safety and efficacy of EUS-guided drainage of PPC using double-pigtail plastic stents in a tertiary hospital.

**METHODS:**

Patients with PPC referred for EUS-guided drainage between May 2015 and December 2019 were included in this case series. The primary endpoint was to evaluate the efficacy (clinical success) and safety (adverse events and mortality) of EUS-guided drainage of PPC. Secondary endpoints included technical success and pseudocyst recurrence.

**RESULTS:**

Eleven patients (mean age, 44.5±18.98 years) were included in this study. The etiologies for PPC were acute biliary pancreatitis, chronic alcoholic pancreatitis, and blunt abdominal trauma. The mean pseudocyst size was 9.4±2.69 cm. The clinical success rate was 91% (10/11). Adverse events occurred in three of 11 patients (27%). There were no cases of mortality. The technical success rate was 100%. Pseudocyst recurrence was identified in one of 11 patients (9%) at 12 weeks after successful clinical drainage and complete pseudocyst resolution.

**CONCLUSION:**

EUS-guided transmural drainage of PPC using double-pigtail plastic stents is safe and effective with high technical and clinical success rates.

## INTRODUCTION

Pancreatic pseudocysts (PPC) are fluid collections with well-defined walls that are rich in amylase and other pancreatic enzymes; they persist for more than 4 weeks inside or around the pancreas and result from pancreatic inflammation and/or a ductal lesion (1). PPC are usually complications of acute or chronic pancreatitis, pancreatic trauma, and pancreatic duct obstruction (2). PPC must be distinguished from other pancreatic fluid collections, including acute peripancreatic fluid collections, acute necrotic collections, walled-off pancreatic necrosis (WOPN), and cystic neoplasms of the pancreas (3).

Most pseudocysts resolve spontaneously and require no endoscopic drainage (2). Indications for drainage include abdominal pain, gastrointestinal obstruction, biliary compression, increased volume documented by computed tomography (CT), and complications such as infection (3,4).

Conventional endoscopic drainage (transmural and/or transpapillary) has been reported since the 1990s, with a success rate of >80% in patients with gastroduodenal bulging or major pancreatic duct disruption (5). EUS-guided drainage is currently the treatment of choice for PPC, with higher safety and success rates than those of other treatment modalities (6,7). EUS-guided drainage has been compared by several authors and proven to be technically superior and safer than conventional endoscopic drainage since it allows drainage in patients with no gastric or duodenal bulging (6,8). Despite controversy regarding the type of prosthesis used for drainage (plastic or metallic), double-pigtail plastic stents are typically used for EUS-guided drainage of PPC (4,9).

This study aimed to evaluate the efficacy and safety of EUS-guided drainage using double-pigtail plastic stents for the treatment of symptomatic PPC at an endoscopy unit in a tertiary center.

## MATERIAL AND METHODS

Between May 2015 and December 2019, 11 patients who were referred to the Endoscopy Unit of Hospital São Paulo, Federal University of São Paulo, Brazil, for PPC and treated with EUS-guided drainage were included in this case series.

Pseudocysts were defined according to the Atlanta classification (10). We included symptomatic patients (abdominal pain and/or complications) with abdominal CT scans demonstrating one or more PPC. The diagnosis of PPC was made based on clinical history, CT scan findings, and appearance on EUS performed immediately before drainage. The exclusion criteria were acute peripancreatic fluid collections, acute necrotic collections, WOPN or other types of pancreatic cysts, patient refusal, pseudocyst distance from the gastroduodenal wall of >1 cm, and irreversible coagulopathy. Patients referred for pseudocyst drainage in whom the presence of necrosis in addition to liquid collection or even WOPN was identified immediately before EUS-guided drainage were considered for endoscopy and/or surgical treatment but not included in this study.

This study was conducted in accordance with the World Medical Association Declaration of Helsinki Ethical Principles and was approved by the Ethics Committee on Human Research (CEP UNIFESP number 0384/2018; CAAE: 87524218.6.0000.5505).

### Procedures

All procedures were performed in patients under conscious sedation or general anesthesia. Broad-spectrum antibiotics (third-generation cephalosporin) were administered to reduce the risk of pseudocyst infection, usually from the day of the procedure until 48h after drainage. All procedures were performed at a tertiary care center by two experienced interventional endoscopists (EDL and FSN) involved in endoscopic retrograde cholangiopancreatography and procedures involving the EUS-guided drainage of PPC. All EUS-guided drainage procedures were performed using a curved linear echoendoscope (EG-53OUT2, Fujifilm Corporation, Saitama, Japan).

**Figure 1:**
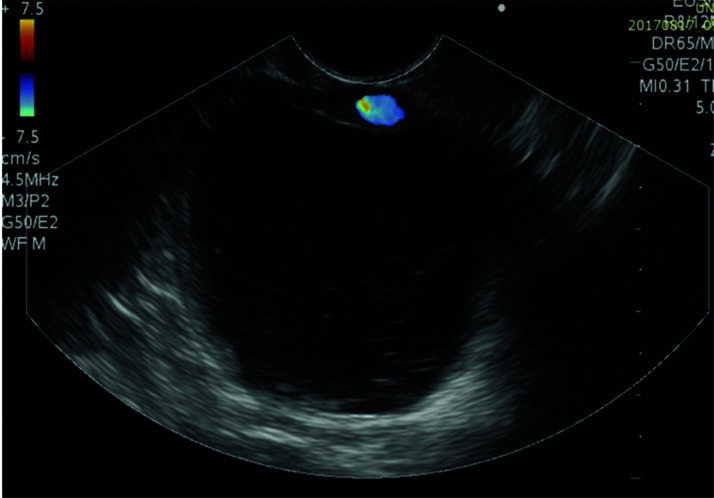
Identification of pancreatic pseudocysts on an endoscopic ultrasound image Doppler ultrasound was used to assess the gastric or duodenal wall for interposed vessels.

**Figure 2:**
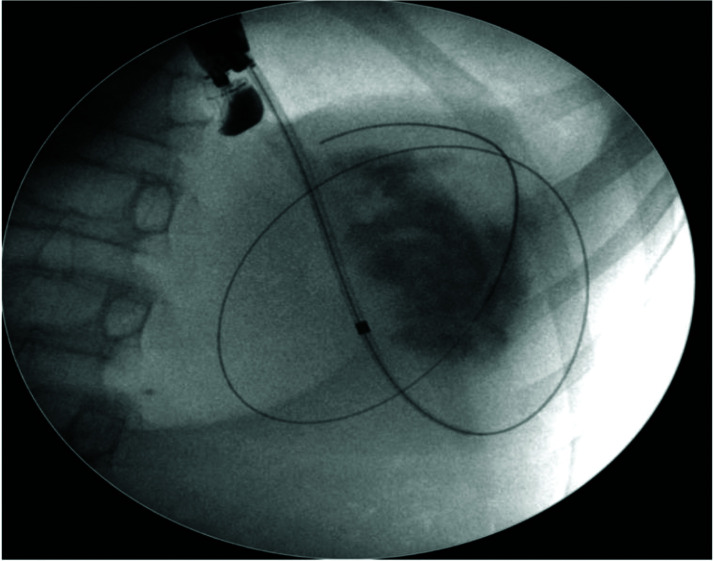
Introduction of a 0.035-inch guidewire by the puncture needle until it formed two loops inside the cyst under fluoroscopic guidance.

**Figure 3:**
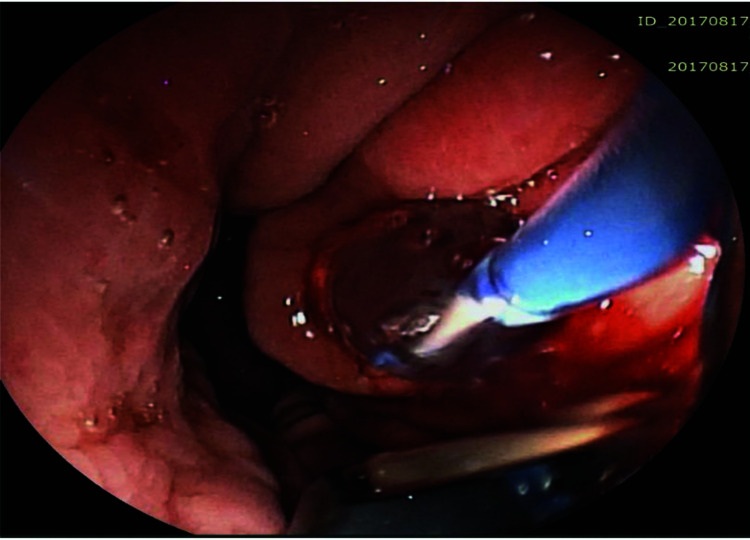
Dilation of the tract using a 10-mm balloon over the wire.

**Figure 4:**
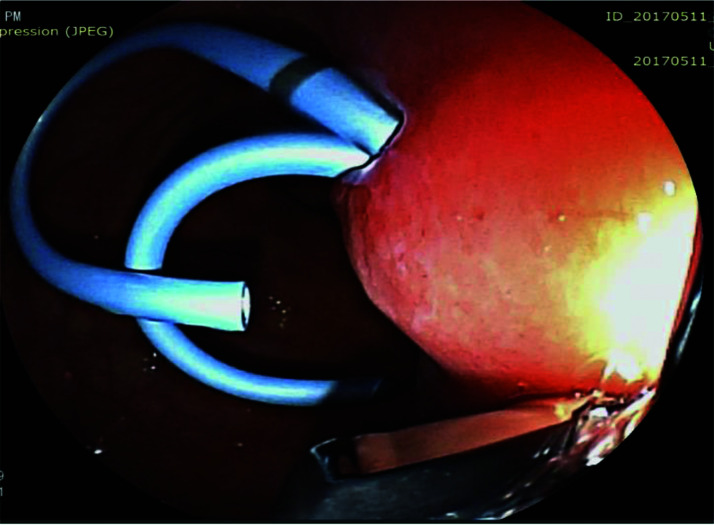
Placement of two double-pigtail stents (10 Fr or 8.5 Fr) to drain the pancreatic pseudocyst.

The pseudocyst was accessed from the stomach or duodenum after the identification of the best puncture point on the gastroduodenal wall up to 1 cm away from the wall. Before puncture, the Doppler function was employed to assess the presence of interposed vessels (Figure 1). The puncture was performed using a 19-gauge needle (Boston Scientific Corporation, Natick, MA, USA). All procedures were performed under fluoroscopic guidance. After the pseudocyst puncture was made, a 0.035-inch guidewire (Jagwire, Boston Scientific Corporation, Natick, MA, USA) was introduced through the needle and coiled within the pseudocyst at least twice (Figure 2). The needle was then removed, and a needle-knife (Sphincterotome Triple Lumen Needle-knife, Boston Scientific Corporation) was used to increase the orifice diameter using electrocoagulation. After this step, the fistula was dilated with a 10- or 12-mm balloon (CRE Balloon Dilatation Catheter, Boston Scientific Corporation) (Figure 3). After the balloon was removed, the guidewire was kept inside the pseudocyst, and a second guidewire was placed inside the cyst. The first double-pigtail plastic stent was inserted, and whenever possible, a second stent was placed (Figure 4). Finally, to prevent aspiration, the stomach was suctioned after pseudocyst drainage. We usually used a 10 Fr or 8.5 Fr double-pigtail plastic stent (Advanix Biliary Stent, Boston Scientific Corporation).

### Endpoints

The primary study endpoint was to evaluate the efficacy and safety of EUS-guided drainage of PPC using double-pigtail plastic stents. Efficacy was evaluated based on clinical success, which was defined as the clinical improvement and resolution of the pseudocyst (decrease in pseudocyst diameter to < 2 cm on CT) at the follow-up performed 4 weeks after drainage. A second drainage procedure was permitted in patients with stent dysfunction (occlusion/migration) up to 4 weeks after the procedure. Treatment failure was defined as the persistence or worsening of symptoms associated with a persistent pseudocyst (pseudocyst diameter >2 cm on CT) 4 weeks after drainage. Safety was evaluated based on adverse events and mortality rates. The complications of EUS-guided drainage of PPC were monitored and stratified as follows: stent migration, stent occlusion, self-limited bleeding related to pseudocyst puncture, pseudocyst infection, perforation, and a false guidewire passage. Secondary endpoints included technical success (defined as the completion of all pseudocyst drainage steps and placement of at least one plastic stent) and pseudocyst recurrence (defined as the return of symptoms and presence of a pseudocyst on CT after complete remission within 6 months of drainage). The stents were removed after pseudocyst resolution was confirmed on CT.

## RESULTS

A total of 11 patients (6 men) with a mean age of 44.5 years (range, 14-79 years) were included in this study. The main etiology was acute biliary pancreatitis (6/11 patients [55%]), followed by chronic alcoholic pancreatitis (3/11 [27%]) and blunt abdominal trauma (2/11 [18%]). Persistent abdominal pain was an indication for EUS-guided drainage in all patients. In addition, one patient had jaundice due to compression of the common bile duct by the pseudocyst.

Most patients (10/11) had only one pseudocyst, while the other patient had three pseudocysts. The mean pseudocyst diameter was 9.4±2.69 cm (range, 5.3-13.5 cm), and the cysts were located in the pancreatic body (46%), pancreatic head (27%), and pancreatic tail (27%). EUS-guided drainage of PPC was performed via the transgastric (10/11) or transduodenal (1/11) route. One patient with a pseudocyst due to chronic pancreatitis was treated with a combination of EUS-guided drainage (transduodenal) and transpapillary drainage of the main pancreatic duct with a plastic stent. A single double-pigtail plastic stent was placed in three patients, while eight patients underwent drainage with two double-pigtail plastic stents. Regarding the number of procedures, nine patients were treated with only one EUS-guided drainage procedure. Two patients underwent a second EUS-guided drainage procedure owing to early stent migration (<4 weeks) and pseudocyst recurrence (>12 weeks).

The technical success rate, characterized by pseudocyst puncture and stent placement, was 100%. Regarding clinical success, 10/11 patients (91%) presented with symptom improvement and pseudocyst resolution at the 6-month follow-up, including the patient who underwent a second EUS-guided drainage procedure owing to early stent migration. Endoscopic treatment was not clinically successful in 1/11 patient (9%). In this patient, abdominal pain did not improve. CT showed a pseudocyst with no reduction in its original size owing to stent occlusion. This patient refused to undergo a second EUS-guided drainage procedure and was referred for percutaneous drainage, which resulted in clinical improvement and pseudocyst resolution.

Pseudocyst recurrence was identified in 1/11 patient (9%) 12 weeks after successful clinical drainage and complete pseudocyst resolution. This patient had a cyst secondary to blunt abdominal trauma, and the recurrence was attributed to disruption of the main pancreatic duct. A second EUS-guided drainage procedure was performed with the placement of two double-pigtail plastic stents; technical and clinical success was achieved. The stents were left for a longer duration, than that after the first placement, resulting in symptom improvement and pseudocyst resolution with no recurrence. The stents were removed after pseudocyst resolution was confirmed by CT (range, 2-6 months). However, it was not possible to determine the average time to stent removal because spontaneous migration after pseudocyst resolution occurred in some patients.

Complications were identified in 3/11 patients (27%). One patient had a false guidewire pathway without any clinical consequences. One patient experienced self-limited bleeding in the gastric wall during pseudocyst puncture and later presented with no pseudocyst resolution, probably because of stent occlusion. This patient (the only one in whom EUS-guided drainage had failed) was referred for percutaneous drainage. The remaining patient showed early stent migration without pseudocyst resolution. The patient was successfully treated with a second EUS-guided drainage procedure. There were no cases of mortality. The patients’ demographic characteristics and details regarding the pseudocysts and the outcomes of EUS-guided drainage of PPC using double-pigtail plastic stents are shown in Tables 1 and 2, respectively.

## DISCUSSION

The results of the present study showed that EUS-guided drainage using double-pigtail plastic stents is effective and safe for the treatment of PPC. Most studies have shown no differences in technical and clinical success rates between the use of metallic and plastic stents (11-15).

In our series, most patients developed pseudocysts after acute biliary pancreatitis; this number was relatively greater than those reported in other studies (16-18). The results of the present study showed high technical and clinical success rates, consistent with those observed in the literature (6,19-22). There is no consensus regarding the number or diameter of plastic stents with respect to EUS-guided drainage of PPC (4). Our intent was to place at least one double-pigtail plastic stent, the definition of technical success (4), and whenever possible, place a second stent side-by-side. However, in our series, it was not possible to place the second stent in three patients owing to technical difficulties (insufficient dilation and loss of the second guidewire, precluding pseudocyst access). However, these patients experienced pseudocyst resolution. However, we agree that whenever possible, placement of the second stent should be attempted to reduce the risk of complications and increase drainage efficiency.

Some complications, such as bleeding, perforation, false guidewire pathway, stent migration or occlusion, and infection, may occur in a few patients. However, most of these cases can be managed clinically and/or endoscopically. Reports of stent occlusion are common in cases of WOPN as well as in cases of infected collections (11,19,21). In our series, no patients had infection after pseudocyst drainage. We believe that some factors may have contributed to this, including the inclusion of only patients with PPC without necrosis and the exclusion of those with WOPN, the use of antibiotics, and the placement of two plastic stents whenever possible. Therefore, EUS-guided drainage using double-pigtail plastic stents is safe, and most complications can be clinically and/or endoscopically managed without the need for surgery or percutaneous intervention.

The recurrence rate of pseudocysts after EUS-guided drainage varies between 7.6% and 13.1%, which could be due to obstruction of the pancreatic ducts (stenosis and/or stone formation) related to chronic pancreatitis or in most cases, after necrotizing biliary pancreatitis or pancreatic trauma (4,23). The recurrence rate in this case series was comparable to that reported in the literature.

A systematic review (23) reported no difference in treatment success rates using double-pigtail plastic stents and lumen-apposing metal stents (LAMS) for PPC (85% *versus* 83%). However, the cost was significantly higher in the LAMS group. Another study compared the double-pigtail plastic stent with self-expandable metal stents and showed that the average procedure time was shorter with the latter option. However, the technical and clinical success rates were 100% and 80%, respectively, in both groups (11).

A systematic review (24) compared the clinical success rates of plastic stents and metal stents for the drainage of WOPN and pseudocysts. Considering patients with pseudocysts alone, the clinical success rate was 98.3% in those with metal stents and 89.4% in those with plastic stents (*p*=0.005). Despite controversy, recent studies recommend using plastic double-pigtail stents as the first-line option for endoscopic drainage and reserving LAMS for special situations, such as cases of non-resolving pseudocysts and pancreatic duct disconnection (9).

This study has some limitations, including a small sample of patients. However, our results are similar to those published by several authors who used EUS-guided drainage for PPC using double-pigtail plastic stents.

## CONCLUSION

In conclusion, in this case series, EUS-guided drainage using double-pigtail plastic stents for PPC was safe and effective with high technical and clinical success rates.

## AUTHOR CONTRIBUTIONS

Sousa GB participated in data acquisition, analysis and interpretation, and in manuscript drafting. Machado RS participated in data acquisition, manuscript drafting and critically review for important intellectual content. Nakao FS participated in data acquisition, analysis and interpretation, manuscript drafting and critically review for important intellectual content. Libera ED participated in the data analysis and interpretation, manuscript critically review for important intellectual content, and was also responsible for the approval of the manuscript final version to be submitted.

## Figures and Tables

**Table 1:** Demographic characteristics of the patients and details of the pseudocysts (N=11).

Characteristic	Value
Male/female ratio	6/5
Mean age, y, ±SD	44.5±18.98
Pseudocyst etiology, n (%)	
Acute biliary pancreatitis	6 (55)
Chronic alcoholic pancreatitis	3 (27)
Blunt abdominal trauma	2 (18)
Mean size of the pseudocysts, cm, ±SD	9.4±2.69
Pseudocyst location, n (%)	
Body	5 (46)
Head	3 (27)
Tail	3 (27)

SD, standard deviation.

**Table 2:** Outcomes of EUS-guided drainage of pancreatic pseudocysts using double-pigtail plastic stents (N=11).

Characteristic	Value
Access route for EUS drainage	
Transgastric	10
Transduodenal	1
Number of double-pigtail plastic stents per patient	
One	8
Two	3
Technical success, n (%)	11 (100)
Clinical success, n (%)	10 (91)
Adverse effects related to EUS drainage, n (%)	3 (27)
False guidewire pathway	1
Self-limited gastric wall bleeding	1
Stent occlusion	1
Pseudocyst recurrence, n (%)	1 (9)
Mortality	0

EUS, endoscopic ultrasound.

## References

[1] Banks PA, Bollen TL, Dervenis C, Gooszen HG, Johnson CD, Sarr MG (2013). Classification of acute pancreatitis - 2012: revision of the Atlanta classification and definitions by international consensus. Gut.

[2] Andalib I, Dawod E, Kahaleh M (2018). Modern Management of Pancreatic Fluid Collections. J Clin Gastroenterol.

[3] Libera ED, Siqueira ES, Morais M, Rohr MR, Brant CQ, Ardengh JC (2000). Pancreatic pseudocysts transpapillary and transmural drainage. HPB Surg.

[4] Dumonceau JM, Delhaye M, Tringali A, Arvanitakis M, Sanchez-Yague A, Vaysse T (2019). Endoscopic treatment of chronic pancreatitis: European Society of Gastrointestinal Endoscopy (ESGE) Guideline - Updated August 2018. Endoscopy.

[5] Barthet M, Lamblin G, Gasmi M, Vitton V, Desjeux A, Grimaud JC (2008). Clinical usefulness of a treatment algorithm for pancreatic pseudocysts. Gastrointest Endosc.

[6] Varadarajulu S, Christein JD, Tamhane A, Drelichman ER, Wilcox CM (2008). Prospective randomized trial comparing EUS and EGD for transmural drainage of pancreatic pseudocysts (with videos). Gastrointest Endosc.

[7] Panamonta N, Ngamruengphong S, Kijsirichareanchai K, Nugent K, Rakvit A (2012). Endoscopic ultrasound-guided versus conventional transmural techniques have comparable treatment outcomes in draining pancreatic pseudocysts. Eur J Gastroenterol Hepatol.

[8] Sauer B, Kahaleh M (2010). Prospective randomized trial comparing EUS and EGD for transmural drainage of pancreatic pseudocysts: a need for a large randomized study. Gastrointest Endosc.

[9] Giovannini M (2018). Endoscopic Ultrasound-Guided Drainage of Pancreatic Fluid Collections. Gastrointest Endosc Clin N Am.

[10] Bradley EL (1993). A clinically based classification system for acute pancreatitis. Summary of the International Symposium on Acute Pancreatitis, Atlanta, Ga, September 11 through 13, 1992. Arch Surg.

[11] Lee BU, Song TJ, Lee SS, Park DH, Seo DW, Lee SK (2014). Newly designed, fully covered metal stents for endoscopic ultrasound (EUS)-guided transmural drainage of peripancreatic fluid collections: a prospective randomized study. Endoscopy.

[12] Lang GD, Fritz C, Bhat T, Das KK, Murad FM, Early DS (2018). EUS-guided drainage of peripancreatic fluid collections with lumen-apposing metal stents and plastic double-pigtail stents: comparison of efficacy and adverse event rates. Gastrointest Endosc.

[13] Sharaiha RZ, DeFilippis EM, Kedia P, Gaidhane M, Boumitri C, Lim HW (2015). Metal versus plastic for pancreatic pseudocyst drainage: clinical outcomes and success. Gastrointest Endosc.

[14] Bang JY, Hasan M, Navaneethan U, Hawes R, Varadarajulu S (2017). Lumen-apposing metal stents (LAMS) for pancreatic fluid collection (PFC) drainage: may not be business as usual. Gut.

[15] Ge N, Hu J, Sun S, Linghu E, Jin Z, Li Z (2017). Endoscopic Ultrasound-guided Pancreatic Pseudocyst Drainage with Lumen-apposing Metal Stents or Plastic Double-pigtail Stents: A Multifactorial Analysis. J Transl Intern Med.

[16] Habashi S, Draganov PV (2009). Pancreatic pseudocyst. World J Gastroenterol.

[17] Aghdassi AA, Mayerle J, Kraft M, Sielenkämper AW, Heidecke CD, Lerch MM (2006). Pancreatic pseudocysts - when and how to treat?. HPB (Oxford).

[18] Cui ML, Kim KH, Kim HG, Han J, Kim H, Cho KB (2014). Incidence, risk factors and clinical course of pancreatic fluid collections in acute pancreatitis. Dig Dis Sci.

[19] Park DH, Lee SS, Moon SH, Choi SY, Jung SW, Seo DW (2009). Endoscopic ultrasound-guided versus conventional transmural drainage for pancreatic pseudocysts: a prospective randomized trial. Endoscopy.

[20] Bang JY, Hasan MK, Navaneethan U, Sutton B, Frandah W, Siddique S (2017). Lumen-apposing metal stents for drainage of pancreatic fluid collections: When and for whom?. Dig Endosc.

[21] Ang TL, Kongkam P, Kwek AB, Orkoonsawat P, Rerknimitr R, Fock KM (2016). A two-center comparative study of plastic and lumen-apposing large diameter self-expandable metallic stents in endoscopic ultrasound-guided drainage of pancreatic fluid collections. Endosc Ultrasound.

[22] Hammad T, Khan MA, Alastal Y, Lee W, Nawras A, Ismail MK (2018). Efficacy and Safety of Lumen-Apposing Metal Stents in Management of Pancreatic Fluid Collections: Are They Better Than Plastic Stents? A Systematic Review and Meta-Analysis. Dig Dis Sci.

[23] Bang JY, Hawes R, Bartolucci A, Varadarajulu S (2015). Efficacy of metal and plastic stents for transmural drainage of pancreatic fluid collections: a systematic review. Dig Endosc.

[24] Saunders R, Ramesh J, Cicconi S, Evans J, Yip VS, Raraty M (2019). A systematic review and meta-analysis of metal versus plastic stents for drainage of pancreatic fluid collections: metal stents are advantageous. Surg Endosc.

